# Multi-Stakeholder Dialogues as Instrument for Design and Qualitative Research in Educational Organisations

**DOI:** 10.1007/978-3-030-55878-9_2

**Published:** 2020-07-15

**Authors:** Christian Helbig, Sandra Hofhues, Bence Lukács

**Affiliations:** 1 Learning, Design and Technology / UNESCO Deputy Chair of Data Science in Higher Education Learning and Teaching, University of Mannheim / Curtin University, Mannheim / Perth, Germany; 2 Department of Education and Social Sciences, University of Cologne, Köln, Germany; 3 Learning, Design & Technology, University of Mannheim, Mannheim, Germany; 4 Human Sciences Faculty, University of Cologne, Cologne, Germany; 5 University of Cologne, Köln, Germany; 6 Department of Education and Social Sciences, University of Cologne, Köln, Germany; 7 University of Passau, Passau, Germany

**Keywords:** Qualitative organisational research, Group discussions, Documentary method

## Abstract

The article focuses on the value of group discussions both as a method of organisational development and as a method of empirical social research. These two perspectives are discussed as a “double meaning”, which often occurs simultaneously in different forms. The concept of “multi-stakeholder dialogues” takes up this challenge. Following on from this, dimensions of the design and research of group discussions will be discussed. The contribution relates to the subproject “Multi-stakeholder Dialogues and Qualitative Evaluation” of the joint project “#ko.vernetzt”. The subproject had the task of accompanying, structuring and researching organisational development in a networked educational institution with dialogue formats. A total of nine dialogues were conducted with different groups of participants, six of which were analysed using qualitative methods. The research perspective is based on a concept of organisations from a praxeological perspective and an understanding of organisational culture as collective conjunctive experience. Thus, the object of qualitative research is the reconstruction of typical modus operandi of the processing of requirements. The results show that structural deficits in educational organisations are reproduced and reinforced by digitisation.

## Introduction

Currently many, if not all, organisations are facing massive and sudden changes to their communication structures and work processes as they grapple with the term *digital transformation* *–* since it is suddenly becoming an absolute necessity (as we are writing this chapter, the outbreak of the novel coronavirus is turning into a global agenda for society, research and education). One of the main questions that arises during the tackling of these challenges is if organisations are just looking for a quick fix, or if they might be able to turn the corner and introduce sustainable transformative procedures.

The challenges of building and maintaining technical and digital infrastructures have been keeping many organisations on their heels, be it with a focus on manufacturing (i.e. Industry 4.0) or on education (e.g. distance education and open education). A question that mostly remained in the background was that of how people would be able to deal with these new and innovative infrastructures, when they eventually arrive through a deliberate implementation process. But as the current situation we are facing shows, simply providing adequate technical and digital infrastructures does not ease the burden properly. Although the immediate needs can be bridged (i.e. keeping classes going virtually), a sustainable transformation of the attitudes and practices requires specific concepts, methods and an entire thought-out process.

In public, as well as political discourse, the technical perspective on digital transformation is given relatively much space (e.g. Altenrath et al. 2020). Thus, digital infrastructures are often described as the basis on which digital transformation would succeed. On the one hand, there is a growing consensus in social science that digital transformation has to be understood as a transformation of individual and collective social practices (i.e. Krotz 2009; Lundby 2014) – with an uncertain outcome: Individual and collective action changes educational organisations and organisational environments, as studies on sociotechnical systems (i.e. Avis 2018) or technostress (i.e. Hassan et al. 2019) show. Whether and to what extent organisations develop depends on themselves, i.e. their set goals and their social practice. On the other hand, organisations and their bureaucracies likewise affect society and social practices. In this light it is necessary to look at people in organisations and organisational learning in the context of digital transformation processes empirically and theoretically, e.g. to ask *how an educational organisation with differentiated educational domains deals with digitisation requirements*. In this way we address those transformation processes that are expressed by collectives and their verbalised practices. To this end, we focus in particular on the importance of dialogue formats both for promoting development processes and for researching organisations.

First, we will present and discuss (methodological) definitions of group discussions by differentiating between two major types – mediating and investigative group discussions. Based on these definitions, we explain design dimensions (Chap. 10.1007/978-3-030-55878-9_3) and empirical dimensions of dialogue formats in learning organisations (Chap. 10.1007/978-3-030-55878-9_4), before we present results from the dialogue formats in the project #ko.vernetzt^1^ (Chap. 10.1007/978-3-030-55878-9_5). After outlining the results, we finalise the article with a conclusion (Chap. 10.1007/978-3-030-55878-9_6). In order to answer our main research question, we will go into more detail about group discussions in the following.

## Double Meaning of Dialogue Formats

If one wishes to study social practice in educational organisations, there are fundamentally different ways of approaching one’s own questions from a research perspective. On the organisational level, a wide repertoire of quantitative and qualitative methods in empirical social research can be used. In the following we therefore present our basic assumptions (Sect. 2.2.1), followed by the introduction of two different perspectives on discussion formats (Sects. 2.2.2 and 2.2.3). At the end of the chapter, we introduce the so-called multi-stakeholder dialogues , which conceptually refer to a combination of organisational research and organisational development (Sect. 2.2.4).

### Framework and Basic Assumptions

If one reflects on the research question, how people approach their challenges in the process of work and what role technology plays in this process, it quickly becomes clear that ways of questioning and observation would be important for clarifying the initial question.

Digitisation, understood as the current surge of mediatisation, is linked to processes of social change – how we communicate and interact with each other (Krotz 2009). These processes of change at all levels of society imply that social science theories about society and social action are only temporarily valid. In our paradigmatic presuppositions, we link to Glaser and Strauss (1969), who emphasise that a theory that may be obsolete, e.g. as a result of social change processes, cannot be refuted by statistical falsification, but by an alternative theory. However, it is not our aim to present an existing theory as outdated, but rather that new readings should enrich theory formation. Accordingly, it is necessary to develop new theories through interpretative and reflective methodologies, e.g. through qualitative research. Furthermore, we are interested in how people interact in an organisation. Here, group discussions have proven to be an appropriate solution for people to talk about their everyday working lives.

For the purposes of the topic, it is important to properly define the term dialogue format. We refer to the international and especially the German discourse on group discussions in empirical research. According to Lamnek (2005, p. 29), the different types of group discussion procedures can be classified according to the respective intention of the findings: In group discussions, which have the goal of collecting information and findings of a content-related nature, or about group dynamic processes, he understands those to be investigative group discussions. Group discussions, which are proposing changes in the behaviour of the respondents, are herein referred to as dialogue formats with a mediating character. Both types are relevant for the reflection of the topic and require more in-depth explanations. Subsequently they are brought together again in the concept of multi-stakeholder dialogues (see Sect. 2.2.4).

This contribution is based on an understanding of research as qualitative research and an understanding of group discussions as interactive-dynamic processes that serve both mediation and investigation.

### Mediating Group Discussions

In the first place, dialogic activities in educational research practice refer to the interaction and cooperation of teachers and students in (formal) learning contexts or in peer-to-peer concepts (e.g. Gilles 2016; Webb 2009). According to Gilles (2016), the cooperative classroom is considered a space for dialogic interactions, while Webb (2009) described the promotion of dialogue as a fundamental task of teachers in classrooms. Focusing on the professional development of teachers, MacPhail et al. (2014) pointed out the importance of learning in communities of practice through cooperation and the exchange of ideas, and Camburn and Han (2017) emphasised the cooperation of teachers as a key element in learning communities.

The Communities of Practice model has received attention, particularly in the context of the discussion on organisational learning (e.g. Argyris and Schön 1978; Wenger 1998). It refers to groups of people who are informally and/or supra-organisationally connected and face similar requirements, i.e. the learning actions in social relationships are in focus (Lave and Wenger 1991). Wenger (1998) characterises communities of practice on the basis of several principles, of which only a few have been selected here:

- Sharing historical roots.
- Having related enterprises.
- Having overlapping styles or discourses.
- Competing for the same resources.
- Specific tools, representation and other artefacts.
- Local lore, shared stories, inside jokes, knowing laughter, jargon and shortcuts to communication as well as the ease of producing new ones^2^ (Wenger 1998, p. 125–127).

Learning thus happens as a relationship between individuals who focus on a shared object. Changing market situations made the model relevant for companies and elevated it to a key concept of corporate development. The related concept of learning organisations is therefore also to be understood strategically and is negotiated as a solution for innovation requirements (Faulstich 2013, p. 192). Argyris and Schön (1978) distinguish three levels of organisational learning: firstly *single-learning* (Simple target-means-result comparison, deviations and discrepancies are perceived and corrected. The goals are rigid, while the means are varied.) (Faulstich 2013, p. 193), secondly *double-loop learning* (Dynamic contexts make it necessary to adapt goals. Learning therefore also includes goal checking.) (ibid.) and thirdly *deutero-learning* (Learning achievements, learning experiences and learning conditions are reflected upon with regard to their promoting and hindering aspects. The aim is to improve learning skills and to increase problem-solving potential.) (ibid.). The focus is always on knowledge in organisations, according to which the organisational knowledge base is activated depending on the situation (“cognitive basis”). This type of knowledge is composed of recognised standards, strategies, views and models of the environment, which together form organisational structures. However, the members of the organisation are permanently forced to interpret and reference these structures. Accordingly, organisations are to be understood here as open, social systems, which develop mechanisms through which they generate the required information, which in turn is interpreted by individuals in communication and interpretation processes (Faulstich 2013, p. 193). These open and broad definitions of learning organisations offer the opportunity to reflect on organisational change and intervention strategies, such as mediating group discussions. It becomes clear that communities of practice go beyond communication and act together on the basis of shared meaning. Learning is thus understood here as a process of habitualisation in a cultural context (Faulstich 2013, p. 196). In the context of these understandings of communities of practice and learning organisations, mediating group discussions present both rationalisation strategies, opportunities for participation and intervention.

In summary, an understanding of group discussions as “mediating” opens a perspective in which organisational development can be promoted in particular through dialogue formats. In the context of digital transformation, this means that organisational development does not take place through the existence or acquisition of technical infrastructure, but rather through the communities of practice and spaces for dialogue. Similar perspectives on organisational development or organisational design can also be found in approaches such as human-centred design (i.e. Magalhaes 2018).

### Investigative Group Discussions

From an empirical perspective, group discussions are used in different contexts since the 1990s. However, methodologically it is necessary to differentiate between interaction-focused and non-interaction-focused surveys. The latter variant particularly includes group methods that do not provide opportunities for interaction (e.g. group interviews) and those that are not initiated by researchers (e.g. natural discussions) (Lamnek 2005). In the following we use the term group discussion exclusively as an interaction-focused method of qualitative social research.

In both the German and international discourse, interaction-focused group discussions are understood as a social interaction space in which discussions between existing groups are generated as naturally and authentically as possible. Since the contributions of the participants refer to common events in the context of their shared life and their shared experience, the views expressed and experiences presented by the other participants could be critically questioned and supplemented. In addition, the existing groups provided a social context in which the attitudes and ideas of the interested persons are formed and developed (Kitzinger 1994). However, the composition of group discussions is not trivial and depends on the research intention. In the Anglo-Saxon discourse on group discussions in particular, the advantages and disadvantages of natural and artificial groups are raised. In both forms of composition in interaction-focused group discussions, however, common characteristics can be identified, which must be weighted differently depending on the research subject (Mäder 2013, p. 26): Group discussions made it possible to get an access to the language, concerns and concepts of the participants. According to Wilkinson (1998a, p. 193), they offer an opportunity “to observe the process of sensemaking” (ibid.), by investigating how opinions are articulated, modified, negotiated and defended (Kitzinger 1994). A special feature of group discussions is seen in the possibility to analyse the interactive processes of the “co-construction of meaning” (Wilkinson 1998b).

Even though the German-language discourse makes hardly any reference to the international discourse, there are parallel occurrences. The positions of the “ Frankfurt Institute for Social Research” in particular are being considered in methodological considerations. Pollock (1955) assumed that opinions on topics of general and public interest do not develop in isolation, but “in constant interaction between the individual and the society” (Pollock 1955, p. 32). Through group discussions, it should be possible to identify partly tabooed and latent opinions. Mangold (1973) went even further and assumed that the articulated statements of members of a group are the product of collective interactions and must therefore be seen exclusively as group opinions. According to Mangold, the group opinion has “in reality already formed among the members of the collective in question” (1973, p. 240) and is merely updated in the group discussion. In recent years, Bohnsack has been linked with the theory formation and empirical reflection of group discussions in Germany. He refers to Pollock and Mangold and assumes that in group discussions collective ascriptions of meaning and significance that exist between representatives of classes or milieus are updated (Bohnsack, 2010).

The previous section has shown that individual statements are not evaluated on the level of what is said; above all, what is said is related to statements of others and, to a certain extent, between the lines various interpretations are explored that result from the given statements. In this process, the interpretation process is usually guided by those passages which have a high density of interactions and metaphors.

### Unifying Concept: Multi-Stakeholder Dialogues

For the methodological reflection of the authors, both perspectives on group discussion – the mediating and the investigative group discussion – are equally important and should be methodically combined here. For this purpose, it is necessary to introduce and modify a concept from organisational development: multi-stakeholder dialogues (MSD). The MSD is a dialogue-oriented method for accompanying organisational development processes in a business-economic sense (Seufert 2013). The aim is to bring together representatives from different areas of work, responsibilities as well as hierarchical levels of an organisation, across tasks and hierarchies, and to identify and reflect on both the potential and challenges of innovations and to develop consensus-based solutions for concrete problems (Dodds and Benson 2013).

In this understanding, MSD largely correspond with mediating group discussions (cf. 2.1). In the cross-hierarchical negotiations, however, communicative and conjunctive stocks of knowledge are also raised, which can be empirically collected and evaluated. Although mediating group discussions correspond in many points with the theoretical paradigms of investigative group discussions (e.g. the self-running nature), it should nevertheless be reflected upon that the context and starting point of the group discussions strongly determine the variant. Accordingly, one variant of group discussions – the mediatory or the investigative – will always dominate (i.e. function as primary), while the other is secondary and becomes a “ by-product”. This means that in mediating group discussions, the qualitative data are a by-product, since the researchers cannot control the direction of the discussion based on the research interest. The same applies to group discussions that are conducted with an exclusively empirical interest: here, the participating group may experience unintended learning processes. This dilemma cannot be completely resolved, but it can be used productively. In the following, we will attempt to describe “dialogical formats” as design and research method for learning organisations.

## Design Dimensions of Dialogue Formats in Learning Organisations

As has been discussed in the previous chapters, each dialogue format has its own specific methodological groundwork. Additionally, when taking into account the topic or the subject-matter for a group discussion (i.e. digital transformation of an organisation), certain design elements can prove to be more suitable. In the context of the project #ko.vernetzt and the implementation of MSD during the developmental process, much of the design was guided by various models centred around innovation, technological diffusion and science (literacy) communication (Eveland 1986; Rogers 2003; Horst 2008).

The four main principles upon which we conceptualised, organised and implemented our dialogue formats, and which are equally important for design and research, can be described as follows:

- *Involvement of all (internal) stakeholders and interest groups:* One of the basic tenets of organisational development (either as a learning organisation generally or through technological implementations specifically) is to involve people from all relevant hierarchical levels and areas of responsibility, since each person has their unique perspective and relationship towards the focus of the implementation (i.e. technology) and their lives (Cochran 1980). This in turn requires hierarchically open structures, or at least the (consciously enabled/designed) opportunity for every stakeholder to (freely) share their opinion and actively partake in the development process itself. Just as corporate stakeholders determine the direction in which an organisation is developing, be it externally in terms of a supplier-customer relationship or internally in terms of organisational development, do front-line workers have to eventually work with the planned implementation while also finding value in it for their own processes. It should be added at this point that the involvement of all stakeholders and interest groups is not accompanied by a general dissolution of hierarchies. Especially in the investigative perspectives (cf. 4), the existence of power and interpretative sovereignty becomes relevant.
- *Multilateral presence dialogues:* MSD at its core presents unique solutions through combining affordances and needs of various theoretical approaches. It is a dialogue-oriented method which can be implemented with or without media (e.g. digital support structures). With the help of cross-task and cross-hierarchical representations, actors of an organisation are to be brought together as stakeholders representing groups at a (round)table and to share and discuss their own perspectives and opinions at eye level. This process can additionally be supported by technological and digital structures, such as enabling the stakeholders to continually provide opinions and ideas, as well as give and receive feedback in between face-to-face sessions (e.g. via an online communication platform). So in our understanding, an MSD is characterised by the fact that it is able to identify and jointly reflect on the potential of organisational development and the challenges of processing technical innovations implicitly.
- *Consensus for description of objectives and goals:* Another goal of the MSD is to develop consensus-based solutions, focusing on concrete problems that are developed at the (round)table. This ensures that the key points and concept papers that emerge from the dialogues are accepted by as many interest groups as possible, as they are not top-down or externally dictated by the (research) partners involved. It is therefore key to have transparent communication structures, which is especially important when designing asynchronous parts within a developmental process (i.e. setting up digital channels). Although methodical and design innovations can be implicitly implemented, since the groundwork and the goals have been developed collaboratively and openly, communicating one’s intention with regard to certain methodical and design choices needs to be made clear to all participants.
- *Dialogue formats as a development and research method:* The duplication of MSD as mediating and investigative group discussion is purposeful for projects between theory and practice, since this ensures that the design project can be systematically recorded and described.

As can be seen in these design dimensions, the most critical aspects of such a process are the central focus on the participants, their needs and ideas (i.e. human-centred design (Magalhaes 2018)), as well as the clear awareness about the iterative nature of the entire dialogue (and development) process (i.e. identifying a problem, creating a solution, testing and improving upon it). These keystones are mostly discussed through the term “design thinking” (Brown 2008) or co-creation within computer science. In our case, dialogue formats represent a conceptual and methodological cross-section by representing the process itself, in addition to functioning as a possible (driver towards a) solution (i.e. iteratively and implicitly changing the work, thinking and communication processes in an organisation).

## Empirical Dimensions of Dialogue Formats

In the perspective of investigative group discussions, as it has been presented here so far (cf. 2.2), empirical dimensions come into focus, which aim at interactive processes in groups and collective experiences. Even though there are various type of approaches to the evaluation of qualitative data, e.g. grounded theory (Glaser and Strauss 1967) or qualitative content analysis (Mayring 2014), the perspective of the “ documentary method ” (in German “Dokumentarische Methode”) is added here. The documentary method can be traced back to Garfinkel (1961) and Mannheim (1964, 1980) and was significantly further developed in Germany by Bohnsack (i.e. 2010). The method has already been tested in the context of various research subjects and fields, including school and classroom research; childhood, youth and family research; media and reception research; and professionalisation research (Nohl 2020). Recently, methodological considerations on “ documentary organisational research” (Amling and Vogd 2017) were published, which can also be used for research questions on the digital transformation of learning organisations.

The central distinction in the documentary method concerns the two levels of pragmatic knowledge according to Mannheim (1980): communicative knowledge and conjunctive knowledge. The communicative knowledge in an utterance is expressed in the explicit meaning contained in the objectivised (linguistic, iconic and performative) means of expression. It contains the interpretations of the actors about their own praxis, but the observer does not gain insight here into the praxis itself. Communicative knowledge remains at the level of theorising about praxis and, as common sense theoretical knowledge, the knowledge about the activity. With the documentary method , Bohnsack proposes a methodical procedure in the interpretative paradigm of empirical social research, with the help of which the conjunctive knowledge behind the communicative knowledge is to be made visible. According to this, the statements of the group members are connected through “collective frameworks of orientations”, which develop in the same way in different groups consisting of members of the same classes or milieus. This also makes it clear which specific characteristics, according to Bohnsack et al. (2010), can form a collection of people into a group: they share common experiences, possibly without knowing each other personally.

Against this theoretical background, the importance of looking at organisations and – in the sense of this anthology – to raise questions about their digital transformation becomes apparent. As Amling and Vogd (2017, p. 16) point out, the basic theoretical references of the documentary method suggest that research-practical selected groups Dialogue formats: may well be equated with milieus. However, organisations are often characterised by the fact that there are heterogeneous, divergent and conflicting milieu contexts. These different value orientations are, for example, dependent on hierarchies and professional socialisation. As a consequence, guidelines, rules and instructions can appear as external frameworks in organisations. In terms of research methodology, this means that both common collective frameworks of orientations and their respective organisational units must be reconstructed as well as the divergent frameworks of orientations and their processing in everyday work. To address this methodological challenge, Nohl (2013) and Mensching (2008) refer to Ortmann (2003) and his perspective on rules. Consequently, the processing of formal rules in organisations results in informal rules, which are understood as regularities of practice. Organisational milieus thus arise when a pool of informal rules is collectively shared by a group. Organisational research structured according to this principle can enable sociological type formation (Nohl 2013, 2017). Here, however, the limits of documentary organisational research become evident, since actions outside of collective structures are excluded and organisations cannot be analysed completely. With regard to digital transformation processes, however, research questions aimed at collective experiences, frameworks of orientations and practices in dealing with the implementation of digital technologies in learning organisations can be addressed.

## Results from the Dialogue Formats in the Project #ko.vernetzt

In the project “multi-stakeholder dialogue and qualitative evaluation” at the University of Cologne, which was conducted within the context of #ko.vernetzt (cf. Bröckling et al. this volume), mediating and investigative dialog formats were tested simultaneously. Thus the aim was both to initiate developmental processes in a learning organisation and to generate empirical results. Hence it was a challenge to create a balance between primary and secondary objectives (cf. Sect. 2.2.3). Due to the application-oriented approach, which was central to the entire project #ko.vernetzt, the focus was on the mediating aspects, while the investigative/empirical perspectives were a by-product. Nevertheless, there have been constant attempts to strengthen empirical positions and to understand research results as opportunities for organisational development as well (although this is always accompanied by simplification). The research perspective focuses on the question of how an educational organisation with differentiated educational domains deals with digitisation requirements.

### Description of the Study

Over a period of 3 years, a total of nine dialogues were conducted with stakeholders of departments of an educational organisation.^3^ Between 5 and 12 employees and managers from all areas of the organisation (e.g. adult educators, teachers, administration, case management) took part in each dialogue. Accordingly, the participants in the discussion are to be understood as quasi-real groups sharing conjunctive knowledge and experience through the organisational context (Kitzinger 1994). The resulting protocols and research results as well as subsequent recommendations were forwarded to the management as quickly as possible. Due to the application-orientation of the project, the topics of the dialogues were developed in an initial dialogue or – in the sense of the methodological framework – worked out based on the communicative knowledge raised in the discussion between the participants. The framework of the initial dialogue, which was set by the researchers, was limited only by the focus on digital transformation in the educational organisation. Due to the character limitation, results can only be presented here in a cursory manner, which lastly leads to summarising considerations.

The issues raised in the interaction between the individuals of the initial dialogue already provided indications of the organisational culture in the context of digital technologies. The following examples are representative for the issues mentioned: “digital teaching”, “digital knowledge management”, “digital organisation of work” and “digital literacy”. In the double meaning of group discussion described in the introduction, these issues are to be understood both as topics of organisational development and as a research focus. The issues raised have not only been discussed since the term digitisation received its current attributions of relevance but also since the emergence of technical infrastructures in organisations (e.g. Wellmann et al. 1996). This is not intended to pursue the narrative that educational organisations are “lagging behind” but rather that traditional challenges in educational organisations are reproduced and reinforced by digitisation. At the same time, these deficits often become visible and workable through the digital transformation that appears as environmental expectations in educational organisations. A systematic look at group discussions that followed the initial dialogue and focused on the issues mentioned provides further indications of practices in the context of digital transformation and the collective framework of the organisation. Against the background of the limited number of cases, a typification of the organisation should be viewed critically. Nevertheless, we dare to make a preliminary description, which therefore requires further concretisation and validation through research. For this purpose, theoretical contrasts have been developed for each of the following dimensions, which we consider realistic and which can characterise other educational organisations in different ways.

### Individualisation of Digitisation

In the group discussions, both external and self-attributions of individual responsibilities in the context of digital transformation processes can be seen repeatedly. These are documented in discussions that are promoted by both managers and employees and end in conclusion.^4^ These individualised practices are framed in particular by discussions about technical purchases and professional training. Decisions on technical purchases and infrastructure are decentralised and are made individually in the various departments of the organisation. As a result, technological resources are unequally distributed within the organisation, and their potential – through equally decentralised communication channels – remains unused. This is also linked to the self-attribution of responsibilities for professional qualification: While few departments in the organisation have experience with “new” technologies^5^ and as a result individual employees develop qualification needs for themselves, uncertainty and resistance grows among employees with less access to technologies.

This individualisation of the technical infrastructures also reinforces the already prevailing practice of treating the acquisition of new knowledge as individualised. In the group discussions, participation in further training measures in connection with digital technologies is made dependent on individual interests, previous experience and the willingness to acquire qualifications. Although this increases the reputation of technology-experienced employees, e.g. digital teaching becomes more difficult without their support, they are also described as “technology freaks”, which increases the individualisation of digitisation.

On the level of the organisation or the departments, the individualisation of digitisation presents itself as a twofold challenge: On the one hand, superordinate implementations of digital structures, e.g. new software, are made more difficult because individual employees have no previous long-term experience with technologies and are not reached through qualifications or training. On the other hand, the resources for enabling organisation-wide qualifications and training are limited, particularly in the education sector.

A theoretical maximum contrast to this collective framework can be seen as centralised and generalised practices of digitisation. Consequently decisions on the acquisition of technologies are made centrally, and qualifications are offered on a mandatory basis to all employees. It is also conceivable that there could be a more democratic collective framework in which as many stakeholders as possible are involved in organisational decision-making processes relating to digital transformation processes.

### Technology-Driven Digitisation

Another recurring pattern that could be reconstructed in the group discussions is an understanding of digitisation that is attached to technical developments. As already mentioned, references to this collective framework can already be found in the (communicative) issues chosen by the participants themselves: Issues such as knowledge management and (media) didactics are not fundamentally renewed by digital technologies. Instead, existing concepts, attitudes and practices are updated or questioned. The reconstruction of the understanding of digitisation in an organisation thus provides less information on the state of digitisation but more on the collective handling of change processes (Helbig and Lukács 2019).

The technology-driven understanding of digitisation, which was worked out here as a collective framework, is documented in group discussions in the way changes within the organisation are described. For example, new software implementations are often not decided on the basis of added value for employees or addressees, but primarily on the basis of environmental expectations and economic factors. Anchor examples in the group discussions that reflect these frameworks document practices where technologies have been acquired because they are described as new and innovative or practices where the implementation of new technologies alone is expected to add value to organisational processes. The discussions in which these practices are documented are not shared equally by all stakeholders – which is often represented in ritual conclusions^6^ – but they document aspects of a technical understanding of digitisation in the organisation, especially when decision-makers represent these practices.

In the context of #ko.vernetzt, this collective framework also became visible in the initiation of organisational development processes, making the double meaning of dialogue procedures described above explicit. In the course of the project, the practice partner criticised the scientific partner that recommendations were too general and heavily focused on cultural aspects of the organisation and very little on the technical infrastructure. The technology focus as a collective frame of orientation is embedded in this criticism, and the organisation demands its reproduction through recommendations.

A theoretical maximum contrast to a technical understanding of digitisation can be a cultural understanding of digitisation. In this understanding, the focus is not on technologies but on everyday practices that are changing due to social change (Krotz 2009). Stalder (2016) offers a perspective on a “ culture of digitality”, which is characterised by referentiality, communality and algorithmicity. This also develops the possibility that private and professional modes of action are moving closer together, as they are not bound to a software, an application or devices.

### Rigid Organisational Culture

The third dimension of this collective framework relates specifically to practices of change in the organisation. This dimension makes particularly evident the methodological separation of communicative and conjunctive knowledge in the documentary method . Thus the cooperation between science and practice partners came about particularly against the background of pressure for change in the educational organisation. On the communicative level, this environmental pressure was translated into an extrinsic motivation to incorporate the social change of digitisation into the structures and practices of the organisation. In the systematic evaluation of the qualitative data as well as in the documentation of organisational development processes, the contradiction between the formal structure and the inner life of the organisation, as is in principle assumed in documentary organisational research (Vogt 2006), became apparent.

In the group discussions with managers in particular, the need for change was discussed, especially at the level of individual qualifications and the acquisition of new technologies (cf. 5.1; 5.2). These topics were also found in the discussions with skilled workers, who criticised above all outdated or missing technologies in everyday working life. Although the need for modern equipment in educational organisations is undisputed, a collective framework for the practice of organisational development, which can be described as “rigid”, is revealed here. The focus is primarily on change processes that can be implemented without resistance from the formal structure of the organisation and those affected.^7^ Deeper aspects of organisational culture, such as communication structures, task distribution and allocation of competences, are excluded. A representative quote for this form of organisational culture is “it has always worked this way”. According to this, practices from the past are still being implemented, even if the current structures irritate these practices. Organisational development is therefore only superficial, while the organisational culture remains rigid.

An example for a theoretical contrast of this framework is embedded in the discourse on “ digital leadership ”. Leadership is understood in contrast to management, which, for example, focuses on higher and long-term goals instead of short- and medium-term ones, promotes people instead of assigning tasks and develops sustainable strategies instead of maintaining the status quo (Sheninger 2019).

## Conclusion

The article started with a reflection on the general transformation process in organisations, which is discussed in the ongoing process of digitisation. It remains to be seen, or rather an empirical question, whether the current corona pandemic, which is affecting educational organisations in particular, will drive digitisation forwards. As shown in this article, dialogue formats understood as group discussions can offer opportunities to promote developments in the context of digital transformation and generate empirical results. The conclusiveness of our experiences with group discussions in organisational development and research to international discourses is still to be discussed, especially against the background of strong paradigmatic and methodological assumptions. In addition, we were concerned with different and interdisciplinary connections – be it in terms of the benefits that empirical research generates in and for society and for organisations, or be it in terms of the results that a project exemplarily produces in relation to organisational research there with close links to digitisation:

The first point picks up on previous discussions about the usefulness of empirical research, such as the articles in the volume “Der Nutzen wird vertagt” (The benefit is postponed) by Reinmann and Kahlert (2007). In their volume numerous educational scientists have already addressed the question of the extent to which educational science itself can both meet scientific criteria and generate practical added value. The focus is also on expected benefits (i.e. Hug et al. 2007). They are often highlighted as (external) environmental expectations in educational organisational research. However, less consideration is given to where these environmental expectations come from. Instead, they refer back to the legitimatory dimension of organisational action (see Altenrath et al. 2020).

Reflecting secondly on strengths and weaknesses of individual methods and aspects for their combination belongs in an article on group discussions, similarly to an empirical method. For application-oriented projects and concepts, there is no doubt that empirical research methods must be used with caution here. This means that not every method is used in research practice and that research methods are not an end in themselves, especially from an application-oriented perspective. As we have shown in our introduction to group discussions, we would like to emphasise that in our comprehension group, discussions are not just focus groups as they are often used in the area of marketing for the purpose of improving individual products. Although group discussions serve the purpose of verbalisation, their empirical content consists mainly of the interpretation, which lies between what is said and the action itself.

From our empirical example, it can thirdly be deduced that – although there is no doubt that a digital transformation process is taking place in educational organisations – this process makes (non-digital) structural deficits transparent and reinforces them. It even condenses the already existing problems in organisations. In dialogue formats carried out in different educational contexts, digitisation is presented – independent of social or scientific discourse – as a metaphor that serves organisational members to describe challenges in action processes, communication and working conditions.

For this reason too, further thought would have to be given to what role participation in research might play. In the near future, it should be considered whether participation means and enables actual participation, or whether existing power relations are reinforced by participation, i.e. whether they are being concealed. At least these are considerations that can be derived from a linked project of #ko.vernetzt: OERlabs (Hofhues and Schiefner-Rohs 2020). According to Reichenbach (2006), participation in research and/or organisational development would continue here and reproduce rather traditional lines. Whether this is precisely what is intended with an instrument such as the MSD remains to be determined in future projects.

## Footnotes

1 #ko.vernetzt was funded from 2017 till 2020 by the Federal Ministry of Education and Research in Germany (funding reference number 01PZ16002A-D).

2 Among these principles, Wenger (1998) also mentions geographical relationships that seem to become increasingly obsolete in the context of digitisation processes.

3 #ko.vernetzt was conducted with a specific practice partner. In the course of the project, the preliminary assumption that several cases can be found in a networked organisation was refuted. In the context of #ko.vernetzt, the organisational hierarchy had such a homogenising effect (cf. Section 2.5.4) that the practice partner could only be understood as one case. Due to the project context, no further cases could be included, so that the typical comparison of minimum and maximum contrasts had to be renounced. The results are therefore to be understood as “dimensions” of organisational communicative and conjunctive knowledge. The particularities of the individual case cannot be worked out without a comparison with other cases.

4 The reconstruction of the organization of discourse thus plays a central role in the documentary method. In order to be able to work out the framework of a passage of conversation, it is examined “how” the participants interact with each other. A conclusion describes the end of a discussion in an inclusive mode. According to this, orientations are found in these discussions that are jointly produced by the collective (Przyborski 2004, p. 96).

5 The description of technologies as *new* is to be understood here as *new* acquisitions in the organisation and as potentially *new* experiences for individuals.

6 A ritual conclusion describes that topics are concluded without reference to the topics themselves. Consequently, a discussion exists in an excluding mode. Accordingly, different and sometimes irreconcilable orientations are expressed in these discussions (Przyborski 2004, p. 216).

7 Here, perspectives of neo-institutionalism can be linked (e.g. Meyer and Rowan 1977).
